# Intrarenal Renin-Angiotensin-System Dysregulation after Kidney Transplantation

**DOI:** 10.1038/s41598-019-46114-x

**Published:** 2019-07-05

**Authors:** Johannes J. Kovarik, Christopher C. Kaltenecker, Chantal Kopecky, Oliver Domenig, Marlies Antlanger, Johannes Werzowa, Farsad Eskandary, Renate Kain, Marko Poglitsch, Sabine Schmaldienst, Georg A. Böhmig, Marcus D. Säemann

**Affiliations:** 1 Department of Internal Medicine III, Clinical Division of Nephrology and Dialysis, Medical University of Vienna, Vienna, Austria; 2 School of Medical Sciences, Faculty of Medicine, University of New South Wales, Sydney, Australia; 3 Attoquant Diagnostics GmbH, Vienna, Austria; 4 Ludwig Boltzmann Institute of Osteology at the Hanusch Hospital, Vienna, Austria; 5 Clinical Institute of Pathology, Medical University of Vienna, Vienna, Austria; 6 Department of Internal Medicine I Kaiser-Franz-Josef-Spital, Vienna, Austria; 7 Department of Medicine VI with Nephrology and Dialysis, Wilhelminen Hospital, Vienna, Austria; 8 Sigmund Freud University, Medical School, Vienna, Austria

**Keywords:** Podocytes, Kidney

## Abstract

Angiotensin-converting enzyme inhibitors (ACEis) are beneficial in patients with chronic kidney disease (CKD). Yet, their clinical effects after kidney transplantation (KTx) remain ambiguous and local renin-angiotensin system (RAS) regulation including the ‘classical’ and ‘alternative’ RAS has not been studied so far. Here, we investigated both systemic and kidney allograft-specific intrarenal RAS using tandem mass-spectrometry in KTx recipients with or without established ACEi therapy (n = 48). Transplant patients were grouped into early (<2 years), intermediate (2–12 years) or late periods after KTx (>12 years). Patients on ACEi displayed lower angiotensin (Ang) II plasma levels (*P* < 0.01) and higher levels of Ang I (*P* < 0.05) and Ang-(1–7) (*P* < 0.05) compared to those without ACEi independent of graft vintage. Substantial intrarenal Ang II synthesis was observed regardless of ACEi therapy. Further, we detected maximal allograft Ang II synthesis in the late transplant vintage group (*P* < 0.005) likely as a consequence of increased allograft chymase activity (*P* < 0.005). Finally, we could identify neprilysin (NEP) as the central enzyme of ‘alternative RAS’ metabolism in kidney allografts. In summary, a progressive increase of chymase-dependent Ang II synthesis reveals a transplant-specific distortion of RAS regulation after KTx with considerable pathogenic and therapeutic implications.

## Introduction

Blockade of the renin-angiotensin system (RAS) with angiotensin-converting enzyme inhibitors (ACEi) or angiotensin-receptor blockers (ARBs) is a critical component of chronic kidney disease (CKD) management. However, benefits of RAS blockade after renal transplantation remain controversial^1,2^ questioning a positive impact on patient and graft survival^3–9^. Hence, recent studies have failed to demonstrate a delay of CKD progression after renal transplantation or reduction of cardiovascular mortality^6,7,10^ in response to RAS blockade.

The reasons for the ineffectiveness of RAS blockade after renal transplantation (KTx) are still unclear. Apart from competing effects including immunosuppression, chronic allograft rejection, or bacterial and viral infections^11^, the intrarenal production and regulation of both angiotensin (Ang) II and Ang-(1–7) could be an important factor for progressive renal and allograft injury^12^. Hence chronic ACE inhibition may be followed by the return of Ang II concentrations to pre-treatment values - a phenomenon known as ‘ACEi-escape’ – raising questions about its long-term effectiveness^13–16^.

A likely candidate to mediate ACEi-escape is the mast cell enzyme chymase cleaving Ang I to Ang II even in the presence of ACE inhibition^15^. Recently, we have shown that chymase rather than ACE is responsible for local Ang II production in human cardiac allografts^17^. Mast cells have also been implicated in interstitial fibrosis in chronic renal allograft rejection^18^, and increased renal chymase activity may be associated with progressive tubulo-interstitial fibrosis^19,20^. Accordingly, in the ‘classical’ RAS pathway, renal Ang II generation in allografts may be facilitated by chymase (and potentially by other serine proteases including cathepsin G and tonin) rather than ACE and thereby remain unaffected by ACEi therapy.

The discovery of an ‘alternative’ RAS pathway counteracting the classical RAS axis has helped to better define the physiological role of the RAS and its potential role in human diseases. In the alternative RAS pathway, Ang I is digested locally by neprilysin (NEP) and prolyl-endopeptidase (PEP) and Ang II is digested by ACE2 and prolyl-carboxypeptidase (PCP) to generate Ang-(1–7), which finally binds to the Mas receptor^21,22^. The balance between the classical and ‘alternative’ RAS pathway can only be assessed by accurate quantification of its individual components. Until recently this was not possible because of the considerable technical challenges like small sample size obtained from biopsies, near-identical amino acid sequences of angiotensins and their very rapid turnover rates. To overcome these challenges, we have developed and validated a multiplex liquid chromatography-tandem mass spectrometry (LC-MS/MS) approach enabling the accurate assessment of angiotensin metabolite levels and their metabolism within blood plasma and tissue biopsies^23–25^.

Here we investigated the role of the intrarenal RAS and the influence of ACEi treatment on systemic angiotensin levels and intrarenal angiotensin metabolism in KTx recipients in a time-dependent manner^26,27^ Using a novel ultrasensitive mass spectrometry-based method, we found a progressive increase of chymase-dependent Ang II synthesis and could identify NEP as central regulator of the alternative RAS after KTx. The potential relevance of our findings for present and future RAS targeting therapies in NTx patients is discussed.

## Results

### ACEi therapy suppresses plasma Ang II and increases Ang-(1–7) in KTx recipients

**Figure 1:**
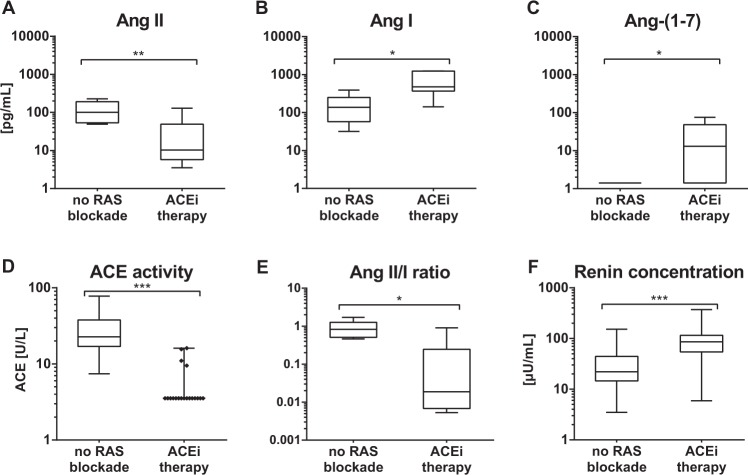
Plasma equilibrium Ang levels. Plasma equilibrium Ang II (**A**), Ang I (**B**) and Ang-(1–7) (**C**) levels were measured in patients without RAS blockade and with ACEi therapy (n = 6 per group). Additionally, ACE activity (**D**), Ang II/Ang I-Ratio (**E**) and renin concentration (**F**) were analyzed in the respective patient groups. Horizontal bars represent median values. Lower limit of quantification: LLOQ. (1 pg/mL (Ang II), 1.3 pg/mL (Ang I), 2 pg/mL (Ang-(1–7)), 5 U/L ACE-acitvity, 1µU/mL (renin)). Time after transplantation (no RAS blockde median 3.0 [0.5–12.6] vs ACEi therapy 7.0 [1.9–13.3]). *P ≤ 0.05, **P ≤ 0.01, ***P ≤ 0.005.

Patients receiving ACEi displayed significantly lower plasma concentrations of Ang II *(P* < *0*.*01)* (Fig. 1A) with no evidence of ACEi escape. By contrast, concentrations of both Ang I *(P* < *0*.*05)* (Fig. 1B) and Ang-(1–7) *(P* < *0*.*05)* (Fig. 1C) were both significantly higher than in patients without RAS blockade. ACEi therapy profoundly suppressed ACE activity *(P* < *0*.*005)* (Fig. 1D), significantly decreased Ang II/Ang I ratio *(P* < *0*.*05)* (Fig. 1E) and increased renin concentrations *(P* < *0*.*005)* (Fig. 1F). These data indicate that ACEi efficiently suppresses systemic Ang II generation in renal transplant patients while upregulating the alternative RAS.

### Tissue RAS enzyme expression

**Figure 2:**
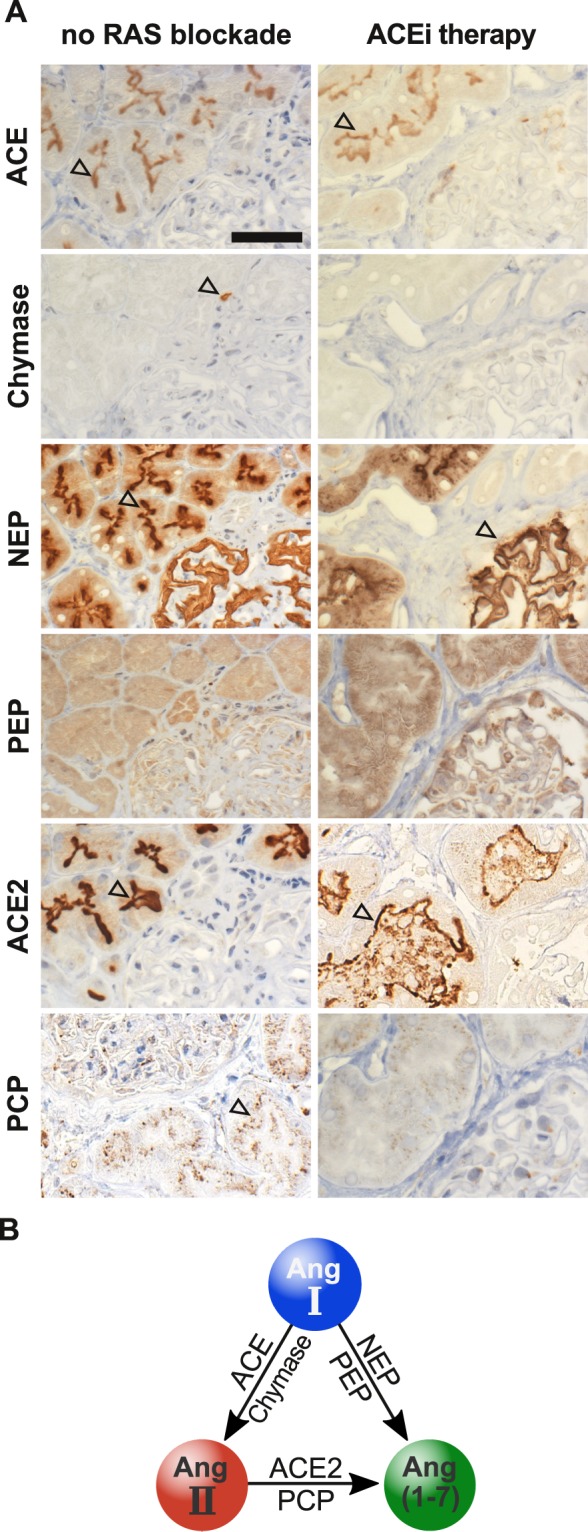
Intrarenal Ang II and Ang-(1–7) forming RAS enzymes. Representative IHC stainings of RAS enzyme expressions of ACE, chymase, NEP, PEP, ACE2 and PCP in kidney biopsies of early biopsy groups with no RAS blockade and with ACEi therapy. (**A**) No differences in immunohistologic expression patterns were observed between different time periods after transplantation except higher incidence of chymase bearing mast cells. Black arrowheads indicate the respective enzymes or cells containing specific enzymes (chymase in mast cells) of representative biopsies. Scale bar equals 50 µm; 400× magnification. Simplified scheme of enzymatic Ang II and Ang-(1–7) synthesis from Ang I and Ang II, respectively (**B**).

We then used immunohistochemistry to determine the cellular distribution of classical and alternative RAS enzymes in renal transplant biopsies of patients with and without ACEi therapy (Fig. 2A). In accordance with results obtained from the Human Protein Atlas (www.proteinatlas.org), NEP was highly expressed at the tubular brush border and within glomeruli, while ACE expression was high in the brush border of proximal tubuli but absent in the glomerula. Chymase was uniquely expressed in interstitial mast cells whilst prolyl-endopeptidase (PEP) showed only a non-specific background signal. ACE2 and prolyl-carboxypeptidase (PCP) showed high tubular expression and enzyme expression patterns were unaffected by ACEi. A schematic overview summarizes the intrarenal enzymatic formation of the principal RAS effectors Ang II and Ang-(1–7) from Ang I and Ang II, respectively (Fig. 2B).

### Angiotensin I and II metabolism within the kidney allograft

**Figure 3:**
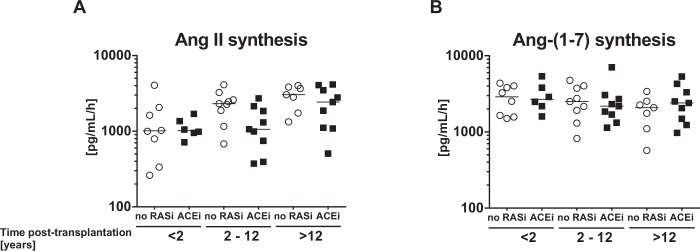
Ang II and Ang-(1–7) synthesis by allograft RAS enzymes. Absolute Ang II synthesis rates from Ang I (**A**) in kidney biopsy homogenates of KTx patients with various graft vintages without RAS blockade (no RASi) or with ACEi therapy were analyzed by mass spectrometry. Similarly, Ang-(1–7) synthesis from Ang I (**B**) was determined.

We quantified enzyme activity for Ang II and Ang-(1–7) synthesis in kidney allografts employing LC-MS/MS (liquid-chromatographic-mass spectrometry/mass spectrometry) after the addition of a standard amount of Ang I to the samples, the natural precursor of both Ang II and Ang-(1–7). Addition of Ang I resulted in profound Ang II formation in all graft vintage groups (Fig. 3A) with the highest production rates found in the late-vintage grafts (Ang II synthesis rates in early-vintage grafts (<2 years): median 1011 [IQR 772–1642] vs. intermediate (2–12 years): 1863 [928–2490] vs. late (>12 years): 2803 [1432–3792] pg/mL/h; *P* < *0*.*005*). By contrast, Ang-(1–7) synthesis rate remained constant throughout all analyzed groups (Fig. 3B) and synthesis of Ang-(1–7) from Ang II (Supplementary Fig. 2A) was similar regardless of allograft vintage or ACEi therapy. Accordingly, increasing allograft vintage is associated with a progressive dysregulation of the classical RAS without obvious changes in the alternative RAS system.

### Graft vintage-dependent alterations of intrarenal chymase and NEP activity

**Figure 4:**
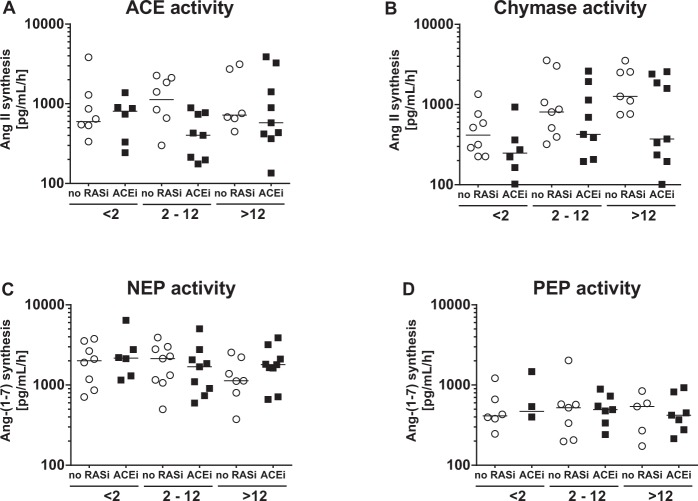
Ang II and Ang-(1–7) synthesis in the transplanted kidney. Contribution of ACE (**A**) and chymase (**B**) activity to form Ang II from Ang I was measured in kidney biopsy homogenates of KTx patients with various graft vintage. After spiking the samples with Ang I as substrate, NEP enzyme activity to form Ang-(1–7) in kidney biopsy homogenates of KTx patient groups by various graft vintage without RAS blockade (no RASi) or with ACEi therapy was analysed by mass spectrometry. (**C**) Similarly, PEP activity was determined (**D**).

Mass spectrometry-based metabolic assays were then used to characterize the contributions of individual enzymes to synthesize RAS effector angiotensins within the kidney allograft. After addition of Ang I as substrate, ACE-dependent Ang II synthesis was constant in all patient groups (Fig. 4A). Chymase activity was moderate in early ( < 2 years) vintage biopsies (median 303 [IQR 224–631] pg/mL/h), but substantially increased in intermediate (2–12 years) (602 [291–1338] pg/mL/h) and the late (>12 years) vintage allograft group (1190 [343–2482] pg/mL/h) (*P* < *0*.*01*); (Fig. 4B).

**Figure 5:**
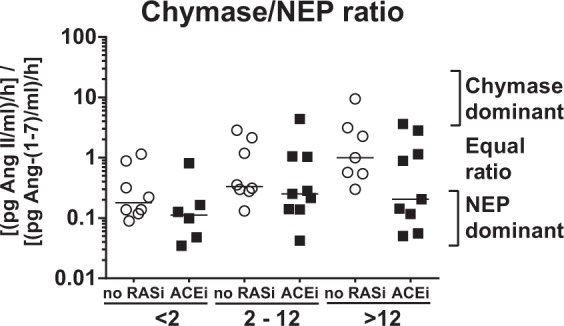
Chymase/NEP-ratio. Calculated Chymase-to-NEP-ratio in kidney biopsies according to graft vintage in patients without RAS blockade (no RASi) or with ACEi therapy.

Remarkably, the overall increase on Ang II formation (Fig. 3A) along with graft-vintage was mediated by chymase rather than ACE (Fig. 4A,B). Analysis of Ang-(1–7) synthesis from Ang I demonstrated sustained NEP activity (median 1807 [IQR 1115–2572] pg/mL/h) in all biopsy groups (Fig. 4C) whereas in comparison prolyl endopeptidase (PEP) mediated Ang-(1–7) formation was clearly lower (463 [321–609] pg/mL/h) (Fig. 4D). Moreover, activity of ACE2, which generates Ang-(1–7) from Ang II was high in all biopsy groups (2206 [890–2982] pg/mL/h, Supplementary Fig. 2B). Analysis of the key metabolites of classical and alternative RAS activity with calculation of the chymase-to-NEP-ratio and depicting of its relative contribution to Ang I turnover demonstrated a significant positive correlation with graft vintage (early 0.14 [0.10–0.29] vs. late 0.73 [0.19–2.39]) *P* < *0*.*05)* (Fig. 5). These data demonstrate that chymase and NEP activity dominate graft vintage-dependent alterations of Ang II and Ang-(1–7) synthesis.

### Accumulation of mast cells in kidney allografts

**Figure 6:**
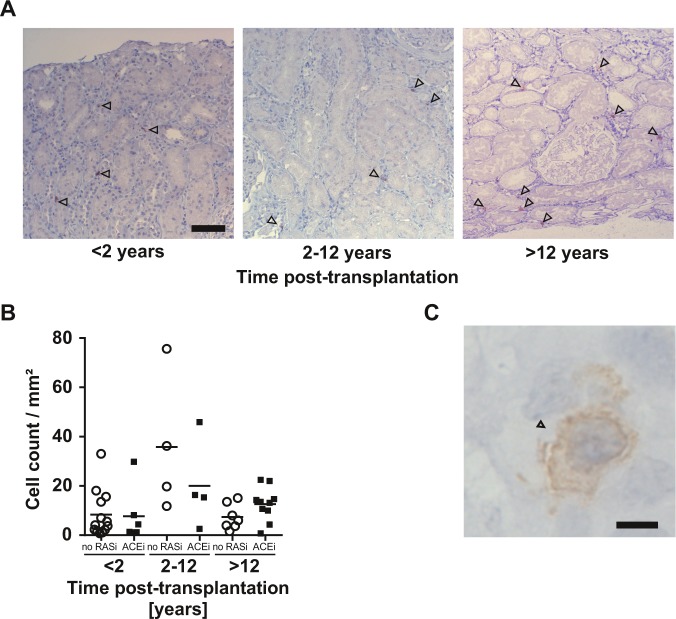
Mast cells in the allograft post KTx. IHC staining of kidney biopsies in both cohorts of analyzed patients revealed accumulation of chymase bearing mast cells (indicated by arrowheads) in allograft biopsies over the years post KTx, independent of ACEi therapy, 100× magnification, scalebar is 50 µm. (**A**) Cell counts displayed higher mast cell numbers in grafts with a graft vintage of two or more years compared to biopsies early after KTx. (**B**) Representative mast cell in allograft biopsy, 630× magnification, scalebar is 5 µm (**C**).

Immunohistochemical staining revealed a graft vintage-dependent accumulation of chymase-bearing mast cells (Fig. 6A). Analyzing chymase-positive mast cells in transplant recipients with ACEi treatment normalized to whole section area, showed a low chymase-positive mast cell count, whereas intermediate and late groups displayed increased mast cell counts (Fig. 6B). Chymase was uniquely expressed in mast cells (Fig. 6C). Similar results were found in patients without RAS blockade (data not shown). These data suggest chymase-bearing mast cells are pivotal to dysregulation of the intrarenal RAS in renal allografts.

## Discussion

In this study, we dissected systemic and local RAS metabolism in KTx recipients across a broad range of allograft vintage by mass spectrometry.

Our data reveal a progressive ACE-independent Ang II synthesis in human allografts over time along with a constantly high NEP enzyme activity. Furthermore, we could identify chymase and NEP as potential novel therapeutic targets for regulating the synthesis of local RAS effectors Ang II and Ang-(1–7). The results provide novel insights into the controversy surrounding RAS blockade in KTx recipients^2,6–9,28^ and identify a molecular pathway that could be the foundation for novel therapeutic approaches to RAS blockade after transplantation.

While the RAS after KTx is constantly affected by various hemodynamic and immunological factors, we have previously shown that the systemic RAS is restored early to physiological levels, i.e. within the first months after KTx indicating that it could present a potential target for pharmacological intervention^29^. However, during the past decade, the focus of interest on the RAS has shifted to the role of the local/tissue RAS in specific tissues^30–32^. Immunohistochemical kidney angiotensinogen (Supplementary Fig. 4) or RAS enzyme expression (Supplementary Fig. 5) and analysis of local RAS activity, by measuring angiotensin metabolism and distinct RAS parameters like urinary angiotensinogen (uAGT)-an index of intrarenal RAS activation that might serve as a useful biomarker, has contributed to centre the interest on intrarenal RAS research in CKD and transplant patients^33–35^.

However, the systemic and graft-specific intrarenal Ang metabolism has not been simultaneously assessed to date. In line with previous results we have shown that KTx patients with ACEi treatment displayed decreased plasma Ang II levels with concomitantly increased renin and Ang I concentrations compared to those without RAS blockade, an effect which was observed independent of transplant vintage^36–38^. Plasma data of patients with no RAS blockade are comparable to our previous results in a healthy control population with normal renal function^39^.

Although we also observed an interindividual variability of plasma Ang levels, therapeutic treatment with ACEi resulted in a distinct and reproducible Ang pattern (low Ang II, high Ang I, high Ang-(1-7)). The resulting high concentrations of circulating Ang I provide a continuous substrate for intrarenal Ang II synthesis generated by chymase or cathepsin G.

We and others have shown that chymase is present in cardiac tissues and plays a dominant role for cardiac Ang II synthesis in heart transplant recipients, whereas ACE only confers a minor functional role^17,40^.

In Sprague-Dawley rats, ACEi therapy suppressed systemic, but not intrarenal Ang II synthesis, demonstrating high activity of local ACE-independent RAS enzyme pathways^41^. However, these findings have not been confirmed in an animal renal transplant models or human KTx recipients so far.

Here we demonstrate that local ACE-independent Ang II synthesis increases with graft vintage, thereby supporting the concept of a time-dependent distortion of intrarenal RAS regulation that could potentially adversely affect allograft outcome. During the first weeks and months after transplantation Ang II synthesis in renal allografts was primarily ACE-mediated closely resembling results of transplant renal biopsies from living donor allografts (Supplementary Fig. 3). Remarkably, while ACE-dependent intrarenal Ang II generation stays stable, chymase-dependent Ang II synthesis gains momentum in grafts with higher graft vintage regardless of graft function. It has to be mentioned that, the contribution of other serine proteases including cathepsin G and tonin to local Ang II formation cannot be formally excluded. Since our patient cohort displayed increasing DSAs with increasing graft vintage, it is tempting to speculate that these antibodies might further trigger allograft damage.

Previous reports implicated mast cells with reduced graft survival and poor functional outcome in KTx recipients^42^. Their molecular transcripts have been linked to renal tissue modifications undergoing nephron decay, remodeling and renal allograft loss^26,42,43^. Moreover, mast cell-associated RNA transcripts correlated with graft age and a high degree of allograft fibrosis underlining an important role of mast cells during the fate of the allograft^26^. The close association between mast cell transcripts and kidney fibrosis implies that therapeutic chymase inhibition could potently attenuate progressive graft damage by directly suppressing intrarenal Ang II synthesis^44^. The ACE-independent chymase mediated Ang II formation and induction of fibrosis might be aggravated in KTx recipients receiving cyclosporine therapy. However, chymase can also induce activation of TGF-β, a major regulator of tissue fibrosis, and matrix metalloproteinase (MMP)-9 activity which seems to be implicated in inflammatory processes independent of the RAS^45^. Although, we may still be far from understanding the exact cellular and molecular mechanisms by which chymase participates in the progression of organ failure, specific chymase inhibitors may emerge as an important tool for defining the role of mast cells and chymase in the pathophysiology of graft damage^46^. Recent data showing that intrarenal Ang II production and the development of hypertension are prevented by selective chymase inhibition support our results of a central contribution of chymase to local allograft Ang II formation^47^. Collectively, chymase inhibition may become an attractive strategy for preventing tissue remodeling and the progression of organ failure, yet further *in vivo* experiments and clinical studies are warranted to support this concept.

Our data further reveal novel principles of alternative RAS regulation in kidney allograft recipients. Previous data indicate that ACE2/Ang-(1–7) axis critically attenuates the detrimental effects of Ang II^38,48^. In line with previous reports, plasma analysis of our KTx cohort revealed high Ang-(1–7) concentrations in patients receiving therapeutic ACEi therapy^39,49^. We have previously reported that NEP rather than ACE2 is the principal enzyme responsible for Ang-(1–7) synthesis generating significant alternative RAS activity in murine and human kidneys^50^. In line, we found that NEP but not ACE2 is the pivotal enzyme for Ang-(1–7) generation in human kidney allografts.

**Figure 7:**
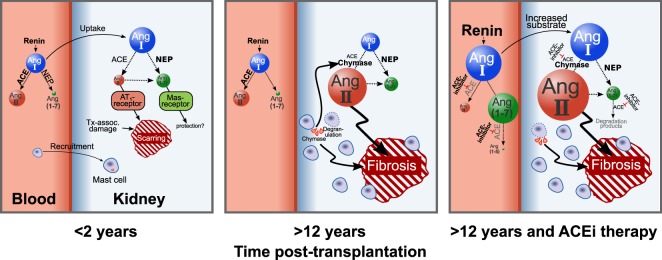
RAS dysregulation after kidney transplantation. Early after KTx (<2years), a moderate ACE-mediated Ang II synthesis occurs in the graft. During the first months after transplantation, Ang-(1–7) synthesis is dominantly mediated by NEP, together with ACE -mediated Ang II synthesis from Ang I. In allografts with higher graft vintage (>12 years), a strong increase of local chymase mediated Ang II synthesis and constant NEP mediated Ang-(1–7) synthesis occurs. In patients with allografts with more than a decade of age, ACEi therapy with subsequent negative feedback and systemic renin upregulation and increased Ang I levels might trigger local chymase mediated Ang II synthesis.

Thus, we propose the following model of intrarenal RAS regulation after KTx: Early after KTx Ang II synthesis within the graft is ACE-mediated, while Ang-(1–7) synthesis is dominantly mediated by NEP from Ang I. In aged allografts a strong increase of local chymase-mediated Ang II synthesis together with constant high NEP mediated Ang-(1–7) synthesis occurs. It is tempting to speculate, that at later phases after transplantation ACEi therapy leads to systemic renin upregulation resulting in increased Ang I levels that might trigger local chymase-mediated Ang II synthesis potentially contributing to further tissue damage (Fig. 7). Given that *in vivo* angiotensin levels are much lower than in the setting of our metabolic assay where exogenous Ang I is abundantly offered to chymase and NEP as a substrate, these enzymes are likely to compete for Ang I *in vivo*. Therefore the observed increase in chymase expression might not only enhance local Ang II formation but also restrict NEP/Ang-(1–7)-mediated alternative RAS activation by exhausting Ang I as a substrate for NEP.

Calculating the chymase-to-NEP-ratio, we found a positive correlation with increased graft age, indicating a profound alteration towards activation of the classical RAS axis in a time-dependent manner after KTx, paralleled by impaired alternative RAS activity. Further studies are necessary to validate this ratio as novel biomarker to interpret the balance between classical and alternative RAS activity as well as the therapeutic efficiency of ACE inhibition.

Observing graft vintage-dependent differences in intrarenal classical and alternative RAS activity strongly supports the concept of a time-dependent adjustment of ACEi therapy. According to our data ACE inhibition might be useful early after KTx, when ACE in the allograft is highly active and ACE-mediated Ang II production is amenable to pharmacological inhibition. However, ACEi might lose their efficacy after several months post transplantation, because chymase and potentially other enzymes will form local Ang II within the allograft. Accordingly, our data emphasize the need for specific studies to re-evaluate the specific role and proper timing of ACEi therapy in stable KTx patients. Even in the presence of ARB therapy, it is conceivable that despite the competitive inhibition of the AT_1_-receptor, Ang II-mediated effects can still occur due to chymase activity. This hypothesis is in line with results of a 5-year study by Ibrahim *et al*. which concluded that treatment with losartan did not lead to a statistically significant reduction in a composite of interstitial expansion or end-stage renal disease in kidney transplant recipients^51^.

It might be further interesting to evaluate the role of novel RAS blockers such as chymase inhibitors and Angiotensin Receptor-Neprilysin Inhibitors (ARNi) in this regard^52,53^. Specifically, ARNi has already shown promising results in heart failure patients by reducing the risk of cardiovascular death and hospitalization rates. Furthermore, in CKD patients ARNi led to a slower rate of decrease in the eGFR and improved cardiovascular outcomes compared to ACEi therapy^54,55^.

The present study has potential limitations: First, the observed progressive and ACEi independent increase of Ang II synthesis in the allografts of our patients may be confounded by individual biopsies that were not studied serially and longitudinally. Second, since we used biopsy homogenates for our analysis, the role of mast cell degranulation and chymase activity could be over-interpreted. Third, the renal biopsies were performed on a per-indication basis (e.g. graft dysfunction, increasing proteinuria) rather than per-protocol (pre-defined time points after KTx). Thus in the setting of graft damage the generalizability of the study findings to stable kidney transplant recipients is formally not possible. Although there is an advantage for analysis of long-term graft vintage, further studies on intrarenal RAS regulation employing protocol biopsies would be advantageous. Additionally, potential gene polymorphisms from the donor kidney could influence our observed results. A varying proportion of patients had a history of T-cell- and antibody-mediated rejection along with a broad variation of DSA positivity. This could have resulted in so far unknown immunological consequences influencing the results of our analysis.

A considerable strength of our study, however, is the highly sensitive mass spectrometry based simultaneous analysis of the classical and alternative RAS metabolites of KTx recipients with a comprehensive range of graft vintages. By identifying the activity of key RAS enzymes like ACE, chymase, NEP and ACE2 with highest accuracy, we could demonstrate a highly distorted local angiotensin metabolism within the human allograft. This innovative approach allowed us to compare systemic and tissue-specific RAS regulation within the same patient, which advantages previously employed techniques prone to methodological limitations such as substrate specificity.

In conclusion, our study demonstrates a profound graft vintage-dependent RAS dysregulation after KTx characterized by an increased Ang II synthesis in aged kidney allografts with chymase as key contributing enzyme. This phenotype is associated with a marked insensitivity towards pharmacological ACE inhibition potentially explaining the inefficiency of RAS blockade in KTx patients. The classical RAS activation is paralleled by an unimpaired alternative RAS activity, where NEP rather than ACE2 is driving Ang-(1–7) production in the kidney allografts. Further studies focusing on chymase and NEP as central regulators of the intrarenal angiotensin metabolism might enable novel therapeutic strategies beyond classical RAS blockade in renal transplant patients.

## Methods

### Study patients

The study was approved by the ethics committee of the Medical University of Vienna (EK 1496/2014) in accordance to the Declaration of Helsinki. Written informed consent was obtained before graft biopsy and blood sampling. This cross-sectional study included 48 KTx recipients (transplantation between 1973 and 2014) scheduled for indication biopsy due to graft dysfunction, proteinuria and/or detection of DSA using single antigen tests [(One Lambda, Canoga Park, CA, USA; MFI threshold: >1000)]. Detailed patient characteristics are summarized in Table 1. No organs/tissues were procured from prisoners. Biopsies were performed between 2014 and 2015 with a median of 55 months (interquartile range (IQR): 8–158) after KTx. For comparative analyses, biopsies were categorized as early (0–2 years; n = 14), intermediate (2–12 years; n = 18) or late post-transplantation (>12 years; n = 16). Twenty-four (50%) of the recipients were on ACEi therapy (enalapril n = 5, ramipril n = 8, lisinopril n = 11; daily dose median at biopsy was 6.3 mg, IQR 5–10) for at least two weeks prior to biopsy (last dose administered at latest two hours before tissue and blood sampling). The remaining 24 patients did not receive any RAS blockade. Additionally biopsy samples of 9 living donors were analysed.

**Table 1:** Baseline patient characteristics.

Biopsy group	early (n = 14)	intermediate (n = 18)	late (n = 16)	P-value
**Epidemiological parameters**				
Age (years)	56 ± 13	51 ± 15	58 ± 10	0.303
Male sex, n (%)	10 (71%)	12 (67%)	9 (56%)	0.668
Systolic blood pressure (mmHg)	134 ± 10	140 ± 13	138 ± 21	0.711
Diastolic blood pressure (mmHg)	76 ± 9	82 ± 14	81 ± 11	0.552
Antihypertensive medication, n (% yes)	13 (93%)	16 (89%)	14 (88%)	0.885
**Tx-associated parameters**				
Time from Tx to biopsy (TxBx, years)	0.3 (0.3)	3.9 (4.0)	14.2 (7.4)	<0.001
Previous transplantation, n (%)	4 (29%)	5 (28%)	2 (13%)	0.478
HLA serotype mismatch, n (A + B + DR)	4 (3)	3 (2)	3 (1)	0.237
Current CDC PRA ≥ 40%, n (% yes)	1/6 (17%)	2/14 (14%)	1/13 (8%)	0.812
Tacrolimus, n (% yes)	13/14 (93%)	10/18 (56%)	1/15 (7%)	<0.001
Cyclosporine A, n (% yes)	0 (0%)	7 (39%)	14 (88%)	<0.001
MMF or MPA, n (% yes)	13/14 (93%)	16/18 (89%)	13/15 (87%)	0.861
Steroids n (% yes)	13/13 (100%)	18/18 (100%)	12/15 (80%)	0.036
Donor specific antibodies, n (% yes)	6 (43%)	13 (72%)	14 (88%)	0.051
Borderline lesions, n (% yes)	1 (7%)	4 (22%)	0 (0%)	0.080
TCMR, n (% yes)	2 (14%)	3 (17%)	0 (0%)	0.276
ABMR, n (% yes)	2 (14%)	5 (28%)	8 (50%)	0.276
IFTA score (ci + ct)	2 (5)	2 (3)	3 (3)	0.150
**Kidney and RAS parameters**				
Creatinine (mg/dL)	2.0 ± 0.7	1.8 ± 0.7	1.8 ± 0.8	0.800
eGFR-MDRD (mL/min/1.73 m²)	37 ± 15	43 ± 21	40 ± 15	0.599
Urinary P/C ratio (mg/g)	326 ± 341	1152 ± 2175	747 ± 1235	0.346
Renin (µU/mL)	23 (71)	44 (67)	73 (83)	0.197
Aldosterone (pg/mL)	134 ± 64	139 ± 94	125 ± 97	0.911
ACE (U/l)	8 (13)	13 (19)	13 (38)	0.373
Sodium (mmol/l)	140 ± 3	140 ± 2	140 ± 2	0.872
Potassium (mmol/l)	4.8 ± 0.5	4.4 ± 0.4	4.6 ± 0.4	0.145

Data are shown as mean ± SD or median (inter-quartile range).

Groups were compared using ANOVA (for continuous variables), Kruskal-Wallis test (for TxBx) or Chi-squared test (for categorial variables). Renin and ACE was log-transformed prior to testing.

ABMR, antibody-mediated rejection; Bx, biopsy; CDC, complement-dependent cytotoxicity; Chronic renal pathology in the interstitium (ci), tubules (ct).

DSA, donor-specific antibody; eGFR, estimated glomerular filtration rate; HLA, human leukocyte antigen; IFTA, interstitial fibrosis and tubular atrophy, MMF, Mycophenolate mofetil, MPA Mycophenolic acid; P/C, protein/creatinine; TCMR, T-cell mediated rejection; Tx, transplantation.

### Plasma collection, sample processing and RAS analyses

At the day of biopsy, patients rested for at least 10 minutes before blood was drawn for laboratory analysis of renin and aldosterone concentration and plasma angiotensin peptide quantification (Attoquant Diagnostics GmbH, Vienna, Austria). Briefly, plasma conditioning for equilibrium analysis was performed at 37 °C followed by stabilization by addition of an enzyme inhibitor cocktail (Attoquant Diagnostics) as described previously^25,39^. We used equilibrated instead of protease inhibitor-stabilized plasma for angiotensin peptide evaluation, since we have previously observed similar qualitative outcomes for biochemical evaluation of the RAS. Stabilized equilibrated samples were further spiked with stable isotope labeled internal standards for each angiotensin metabolite at a concentration of 200 pg/ml. The samples then underwent C18 based solid-phase extraction following standard protocol (Waters, Milford, MA, USA). The eluted samples were evaporated to dryness under a filtered, pre-warmed steady stream of nitrogen gas, reconstituted in 10% acetonitrile/0.1% formic acid and subjected to LC-MS/MS analysis using a reversed phase analytical column (Acquity UPLC BEH C18 Column, 1.7 µm, 2.1 mm × 50 mm, Waters, Milford, MA, USA) operating in line with a Xevo TQ-S triple quadruple mass spectrometer (Waters, Milford, MA, USA). Component A consisted of water with 0.1% formic acid, while Component B was acetonitrile with 0.1% formic acid. A gradient program was used, where the concentration of component B was kept at 5% for 0.5 min initially and increased to 50% over 4 min. Component B was further increased to 95% for 1 min and then returned to 5% for 1 min. At least two different mass transitions were measured per analyte and internal standard signals were used to correct for matrix effects and peptide recovery of the sample preparations procedure for each angiotensin metabolite in each individual sample. Analyte concentrations were calculated by relating endogenous peptide signals to calibration curve, provided that the integrated signal exceeded a signal-to-noise ratio of 10. Inter-assay coefficients of variation (CVs) for Ang I, Ang II and Ang-(1-7) were 10.2%, 6.1% and 7.2% respectively, while the intra-assay CVs were 8.6%, 4.4% and 5.4% respectively.

### Kidney transplant biopsies

Allograft biopsies were performed with ultrasound guidance using a 16 G needle (two cores per biopsy). Within two minutes, cores were evaluated and a small fragment (2 mm length) of one core was separated, snap-frozen in liquid nitrogen and stored at −80 °C for RAS metabolic assay. The remaining biopsy material was formalin-fixed and paraffin-embedded for histomorphological and immunohistochemical assessment.

For routine evaluation of allograft morphology, formalin-fixed paraffin sections were stained according to standard methodology including immunohistochemical C4d staining using a polyclonal anti-C4d antibody (BI-RC4D, Biomedica, Vienna, Austria). Rejection subtypes or individual single lesions were classified and/or scored according to the recent Banff updates^56^. For RAS metabolic assay, frozen biopsy tissue was homogenized in phosphate-buffered saline (PBS) using low-energy sonication. RAS enzyme assay specificity of metabolic assays was tested using selected RAS enzyme inhibitors and recombinant human enzymes (Supplementary Fig. 1).

The *ex vivo* metabolism of added Ang I or Ang II was determined in tissue homogenates after incubation at 37 °C in the presence or absence of enzyme inhibitors of Angiotensin-Converting-Enzyme (ACEi): Lisinopril (10 µM); prolyl-endopeptidase (PEPi)/prolyl-carboxypeptidase (PCPi): Z-Pro-prolinal (ZPP, 20 µM); neprilysin (NEPi): DL-thiorphan (100 µM); chymase (CHYi): chymostatin (10 µM, all Sigma-Aldrich, Munich, Germany); Angiotensin-Converting-Enzyme 2 (ACE2i): MLN-4760 (10 µM, Merck-Millipore, Darmstadt, Germany). Aminopeptidase inhibitor (10 µM, Sigma-Aldrich, Munich, Germany) was added to all samples. Angiotensin measurement was performed as described previously^23,24^. For immunohistochemical detection of RAS enzymes, antigens on formalin-fixed paraffin-embedded sections (4 µm) were unmasked by autoclaving, endogenous peroxidase was blocked (3% H_2_O_2_) and slides were incubated for one hour in primary antibody against RAS enzymes (ACE and PCP, PEP, Sigma-Aldrich, Munich, Germany; ACE2, R&D Systems, Minneapolis; Chymase, Abcam, Cambridge, Massachusetts, NEP, Novocastra, UK), followed by polymer-kit treatment and diaminobenzidine development (all Thermo Fisher Scientific). Slides were haematoxylin-counterstained (Merck-Millipore) and photographed (JENOPTIK ProgRes C12plus, Jena, Germany). Chymase slides double stained with c-Kit (Dako, Agilent Technologies, CA) were used for counting double-positive cells. Total biopsy area was computed using photomicrographs and cell count was normalized to biopsy area. At least two slides of different cutting depths were examined per subject to minimise sampling error.

### Statistical analyses

Normally distributed variables (verified by visualization; renin levels were log-transformed prior to testing) were compared using ANOVA. Fisher’s exact test was applied for dichotomous variables. Angiotensin levels were log-transformed and compared using an unpaired two-sided *t*-test. Linear trend analysis of angiotensin synthesis rates were tested using repeated measures ANOVA and Šidák correction for multiple testing, whereas each group was compared against control incubation without enzyme blockade. A P-value ≤ 0.05 was considered statistically significant. Values below the lower limit of quantification (LLOQ) were substituted by LLOQ/Sqrt(2) for statistical calculations as described previously^57^. GNU PSPP 0.11.4 was used for statistical analysis and GraphPad Prism 6.05 (GraphPad Software, CA) was used for data representation.

## Supplementary information

Supplementary information

## Data Availability

The data that support the findings of this study are available from the corresponding author, [M.D.S.], upon reasonable request.
