# Supplementary material for: Intrarenal Renin-Angiotensin-System Dysregulation after Kidney Transplantation
Source: Sci Rep. 2019 Jul 5;9:9762. doi: 10.1038/s41598-019-46114-x (PMC6611786; doi:10.1038/s41598-019-46114-x)
Supplement: Supplementary file 1 — Supplementary information [file 41598_2019_46114_MOESM1_ESM.pdf]

Intrarenal Renin-Angiotensin-System Dysregulation after Kidney Transplantation

Johannes J. Kovarik[1], Christopher C. Kaltenecker[1], Chantal Kopecky[1,2], Oliver Domenig[1,3], Marlies Antlanger[1], Johannes Werzowa[4], Farsad Eskandary[1], Renate Kain[5], Marko Poglitsch[3], Sabine Schmaldienst[6], Georg A. Böhmig[1], Marcus D. Säemann*[7,8]

Supporting information

Supplementary methods:

Specific activity of each RAS enzyme was calculated by determining the inhibitor sensitive fraction (control minus inhibitor) of product synthesis related to control [(pg/mL)/h, Ang II or Ang-(1-7)]. First, the selectivity of enzyme inhibitors in biopsy homogenates was measured in pooled biopsy samples of all investigated transplant groups. These assays were performed similar to individual samples with additional recombinant human enzymes. The increased in Ang II synthesis induced by addition of recombinant human (rh) ACE was quantitatively blocked by addition of ACEi. Similarly, rhChymase dependent Ang II synthesis was inhibited by chymase inhibitor (CHYi). ACEi did not block rhChymase, while CHYi showed no inhibitory capacity for rhACE in terms of Ang II synthesis **(Supplementary Fig. 1)**.

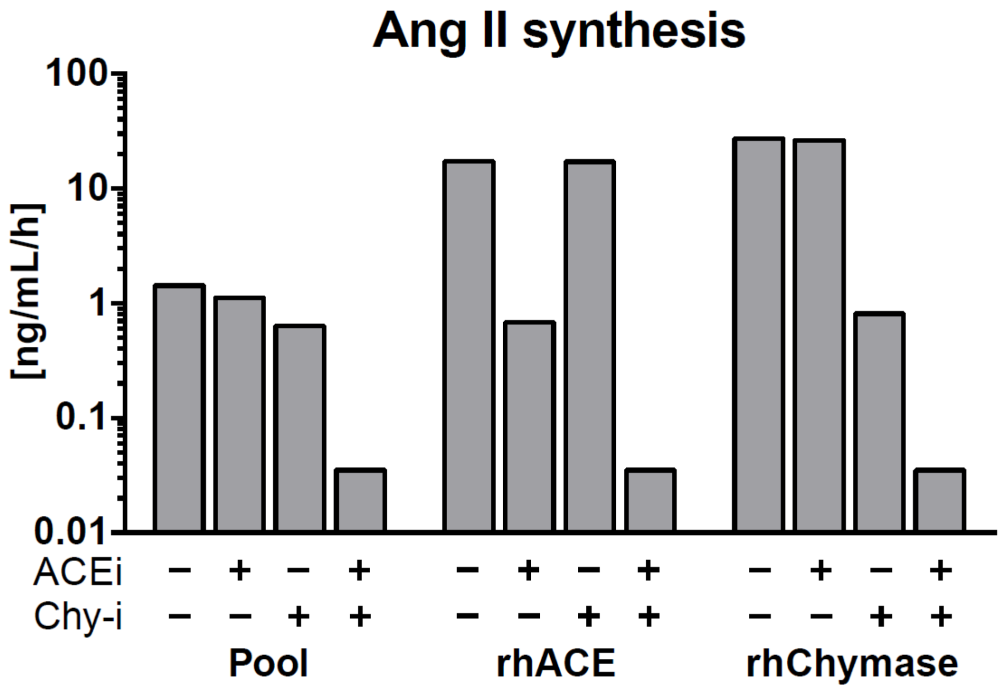

Supplementary Fig. 1: Selectivity of specific RAS enzyme inhibitors used for renal metabolic assays

Ang I was spiked to tissue homogenates and Ang II synthesis was measured. A pool of samples was challenged with rhACE (100ng/ml) and rhChymase (20ng/ml). Ang II synthesis was measured baseline (control) and in the presence or absence after further in vitro challenge with ACEi lisinopril, CHYi chymostatin or combination.

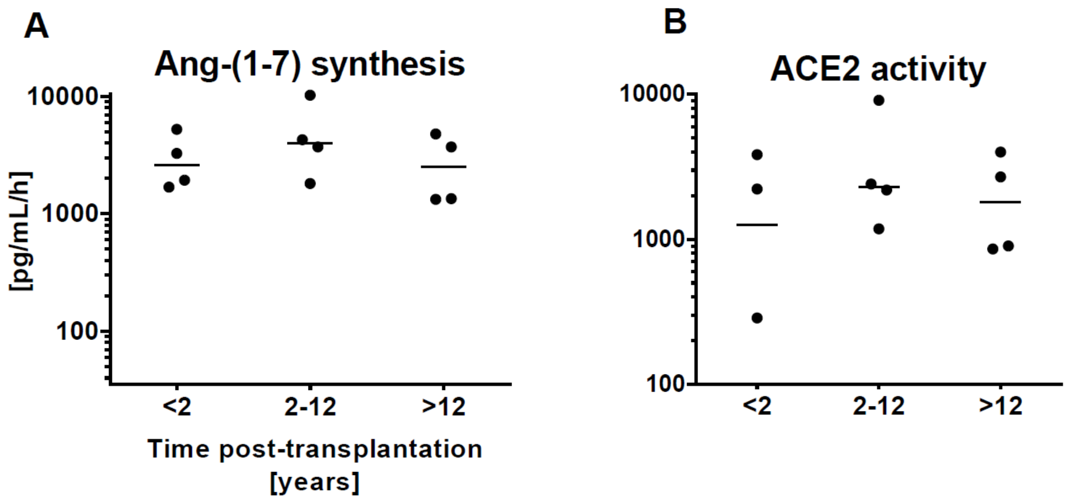

Supplementary Fig. 2: Ang II to Ang-(1-7) turnover

After spiking samples with Ang II as substrate to determine Ang II to Ang-(1-7) turnover (A) and ACE2 enzyme activity to generate Ang-(1-7) was analysed by mass spectrometry (B). (n=4 per graft vintage group). No difference between patients without RAS blockade and with ACEi therapy was observed.

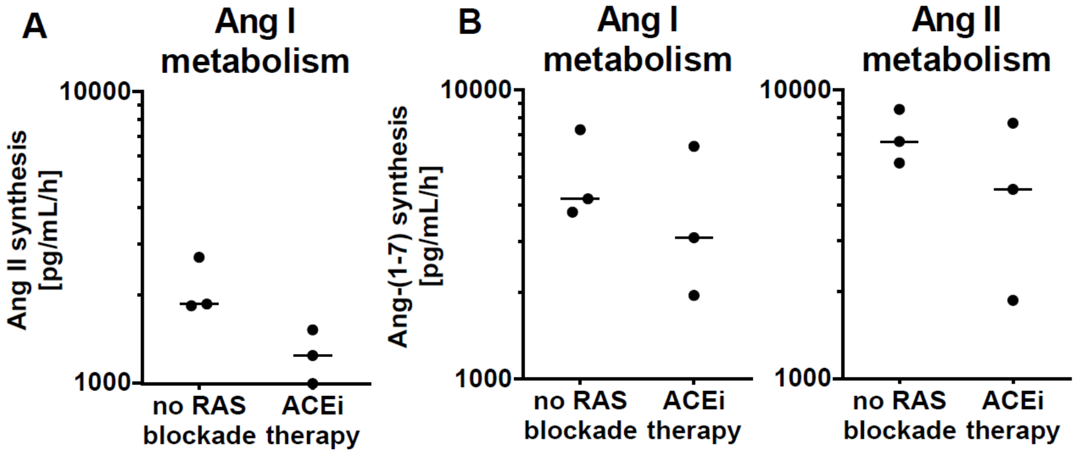

Supplementary Fig. 3: Ang II and Ang-(1-7) synthesis in kidney tissue of living donors

Absolute Ang II synthesis rates from Ang I (A) in kidney biopsy homogenates of healthy living donors by mass spectrometry. Similarly, Ang-(1-7) synthesis from Ang I and Ang II (B) was determined. (n=3 per group).

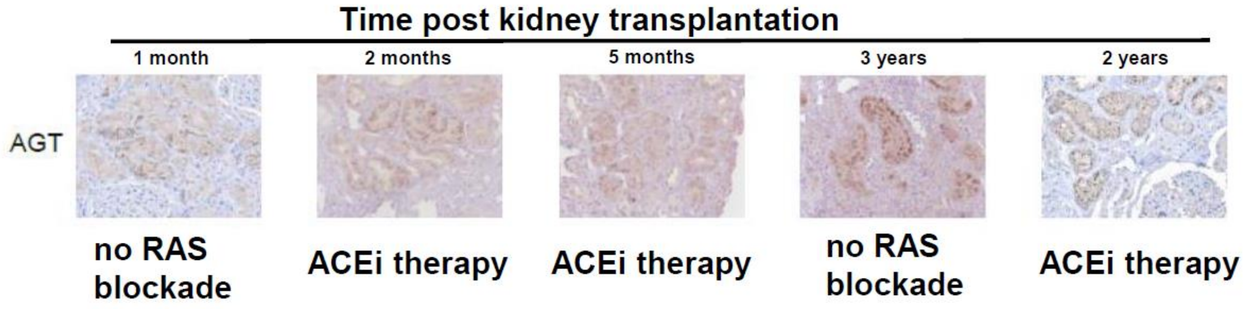

Supplementary Fig. 4: Time course of IHC stainings of angiotensinogen in kidney allografts. (n=5). Both groups showed similar expression patterns. Scale bar equals 50µm; 400x magnification.

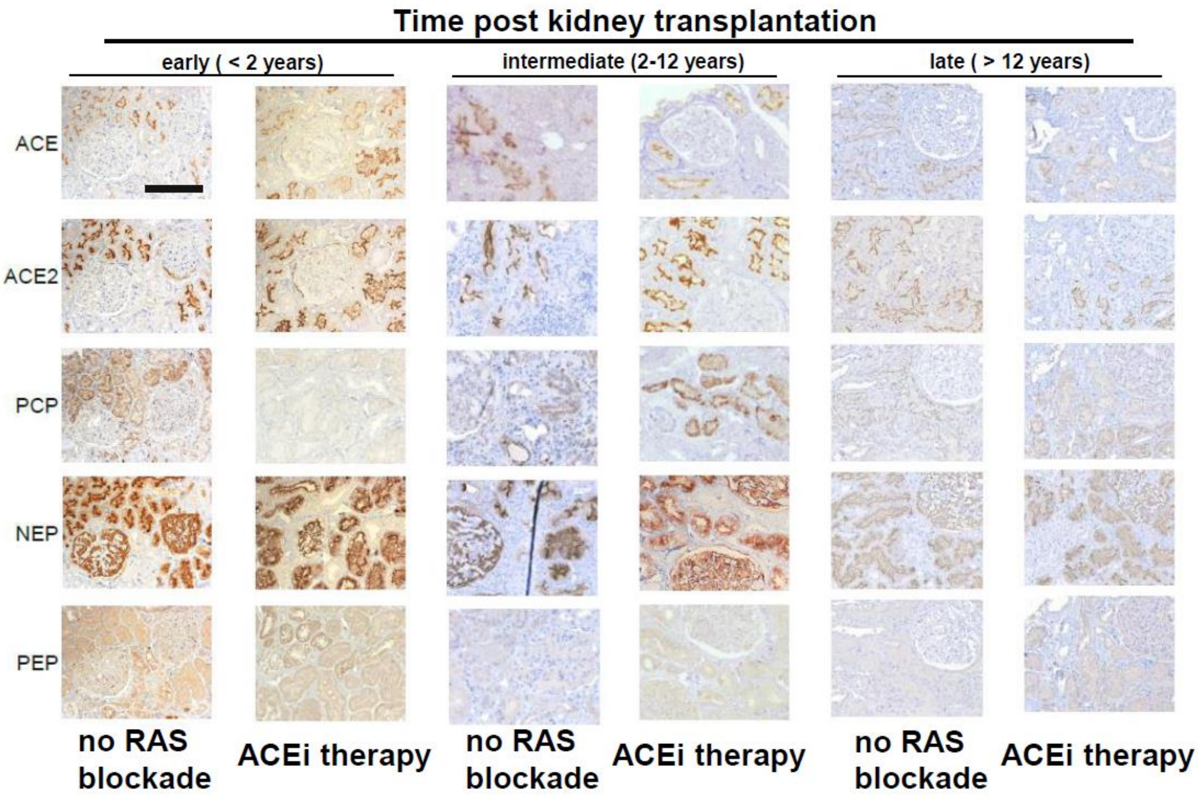

Supplementary Fig. 5: Time course of IHC stainings of RAS enzyme expressions of ACE, NEP, PEP, ACE2 and PCP in kidney biopsies of early, intermediate and late biopsy groups with no RAS blockade and with ACEi therapy. Enzyme expressions were coherent across the respective groups.

Scale bar equals 50µm; 400x magnification.
